# Gait alterations in patients with adult spinal deformity

**DOI:** 10.1016/j.xnsj.2023.100306

**Published:** 2023-12-30

**Authors:** Stephanie M.D. Huysmans, Rachel Senden, Eva Jacobs, Paul J.B. Willems, Rik G.J. Marcellis, Mark van den Boogaart, Kenneth Meijer, Paul C. Willems

**Affiliations:** a Department of Orthopedic Surgery and Research School CAPHRI (Care and Public Health Research Institute), Maastricht University Medical Center+ (MUMC+), Maastricht, the Netherlands; b Department of Physiotherapy, Maastricht University Medical Center+ (MUMC+), Maastricht, the Netherlands; c Department of Nutrition and Movement Sciences, NUTRIM School of Nutrition and Translational Research in Metabolism (MUMC+), the Netherlands

**Keywords:** Adult spinal deformity, 3D gait analysis, Sagittal alignment, Spine, Trunk tilt, Gait characteristics, Spatiotemporal parameters, Joint kinematics, Matched control group, SPM analyses

## Abstract

**Background:**

Adult spinal deformity patients (ASD) experience altered spinal alignment affecting spatiotemporal parameters and joint kinematics. Differences in spinal deformity between patients with symptomatic idiopathic scoliosis (ID-ASD) and patients with “de novo” scoliosis (DN-ASD) may affect gait characteristics differently. This study aims to compare gait characteristics between ID-ASD, DN-ASD, and asymptomatic healthy matched controls.

**Methods:**

In this observational case-control study, ID-ASD (n = 24) and DN-ASD (n = 26) patients visiting the out-patient spine clinic and scheduled for long-segment spinal fusion were included. Patients were matched, based on age, gender, leg length and BMI, with asymptomatic healthy controls. Gait was measured at comfortable walking speed on an instrumented treadmill with 3D motion capture system. Trunk, pelvic and lower extremities range of motion (ROM) and spatiotemporal parameters (SPT) are presented as median (first and thirds quartile). Independent t-test or Mann–Whitney U test was used to compare ID-ASD, DN-ASD and controls. Statistical Parametric Mapping (independent t-test) was used to compare 3D joint kinematics.

**Results:**

DN-ASD patients walk with increased anterior trunk tilt during the whole gait cycle compared with ID-ASD patients and controls. ID-ASD walk with decreased trunk lateroflexion compared with DN-ASD and controls. DN-ASD showed decreased pelvic obliquity and -rotation, increased knee flexion, and decreased ankle plantar flexion. ID-ASD and DN-ASD displayed decreased trunk, pelvic and lower extremity ROM compared with controls, but increased pelvic tilt ROM. ID-ASD patients walked with comparable SPT to controls, whereas DN-ASD patients walked significantly slower with corresponding changes in SPT and wider steps.

**Conclusions:**

DN-ASD patients exhibit distinct alterations in SPT and kinematic gait characteristics compared with ID-ASD and controls. These alterations seem to be predominantly influenced by sagittal spinal malalignment and kinematic findings in ASD patients should not be generalized as such, but always be interpreted with consideration for the nature of the ASD.

## Highlights

• Sagittal spinal malalignment influences alteration in gait characteristics.
• Type of spinal deformity should be considered when interpreting gait parameters.
• Patients with sagittal spinal malalignment walk slower than asymptomatic controls.
• Statistical parametric mapping enables comparison of joint kinematics.
• 3D gait analysis can complement current standard of care.

## Background

Adult spinal deformity (ASD) disrupts the normal alignment of the spine and can cause changes in posture and gait pattern, leading to functional impairments, decreased mobility and quality of life [1]. To counteract sagittal spinal malalignment during standing, ASD patients compensate by thoracic hypokyphosis, reduced lumbar lordosis, posterior pelvic tilt and/or increased hip and knee flexion along with increased ankle dorsiflexion to preserve a horizontal gaze and maintain stability [1,2]. However, it has been shown that these compensatory mechanisms are lost during walking and that the disbalance as a result of the spinal malalignment in ASD patients strongly influences gait parameters and leading to deviation gait patterns [[3], [4], [5], [6],^7^]. Previous studies using 3-dimensional gait analysis in patients with ASD reported altered spatiotemporal parameters, such as a slower walking speed and step cadence with shorter steps in patients with ASD compared with healthy controls [[8], [9], [10], [11], [12], [13], [14], [15], [16], [17], [18]]. Studies investigating kinematics in patients with ASD reported an increased anterior trunk tilt [4,7,19,20], increased anterior pelvic tilt [4,19,20], and consequently more flexed hips and knees [[5], [6], [7]] as compared with healthy controls, in an attempt to keep the center of mass within the base of support. Furthermore, no studies investigated the effect of ASD on gait alterations in the frontal plane with regards to trunk and pelvic motion, although it has previously been reported that spinal deformity in the frontal plane is related to pain and dysfunction in patients with ASD [21].

Symptomatic idiopathic scoliosis (ID-ASD) patients with progression of adolescent idiopathic scoliosis (AIS) typically experience onset of symptoms at a younger age and exhibit normal sagittal alignment on static radiographs [22,23]. However, they often display postural malalignment in the frontal plane. For these patients, surgical intervention may be recommended to prevent further deterioration and associated symptoms. Patients with “de novo” ASD (DN-ASD), who tend to experience symptoms at an older age, show a more pronounced sagittal malalignment. Persistent and severe back pain, along with impaired mobility resulting in difficulty in walking or maintaining an upright posture due to the deformity, can be an indication for surgery [5,7,24].

When conservative measures prove to be insufficient in addressing the symptoms and functional limitations associated with the spinal deformity, a therapeutic option for these patients is spinal fusion surgery. The goals of surgery are to alleviate pain, improve spinal alignment, restore neurological function, and enhance overall mobility by fusing affected vertebrae together to correct the misalignment and stabilize the spine. Instrumentation such as rods, screws, and other devices may be used to support the spine during the fusion process.

This study aims to investigate alterations in gait characteristics of ASD patients compared with asymptomatic healthy controls, while taking into account differences in the origin and nature of adult spinal deformity between 2 patient groups: those with symptomatic idiopathic scoliosis with progression of adolescent idiopathic scoliosis, and those with “de novo” ASD.

## Methods

### Subject sample

In this observational retrospective case-control study, patients who were scheduled for long segment spinal fusion surgery of 4 segments or more between March 2017 and October 2021 were reviewed. Patients aged 18 years or older that with available 3-dimensional gait analyses prior to surgery were included. Exclusion criteria were active cancer, body weight exceeding the equipment rating for the treadmill (135 kg), inability to walk, or mental disability. Three-dimensional gait analyses was performed as part of standard care. Patients were measured within 6 months (range 1–190 days) prior to surgery. This study was approved by the local Medical Ethics Committee. Demographic variables including sex (male or female), age at the time of the gait analysis, weight (kg), leg length (m), height (m) and BMI (kg/m^2^) were recorded. Medical records were examined to identify patients with a history of total hip or knee replacement surgeries. For ASD patients, the indication for spinal fusion, symptomatic idiopathic scoliosis (ID-ASD) or “de novo” scoliosis (DN-ASD) was recorded.

Each ASD patient was matched with an asymptomatic healthy control subject based on age, gender, leg length and BMI. The healthy controls were selected from a reference database. The controls had no self-reported medical history resulting in walking difficulties, balance problems affecting daily activities, spinal deformity, and could walk for at least 30 minutes without assistance.

### Procedure

#### Gait analysis

**Fig. 1:**
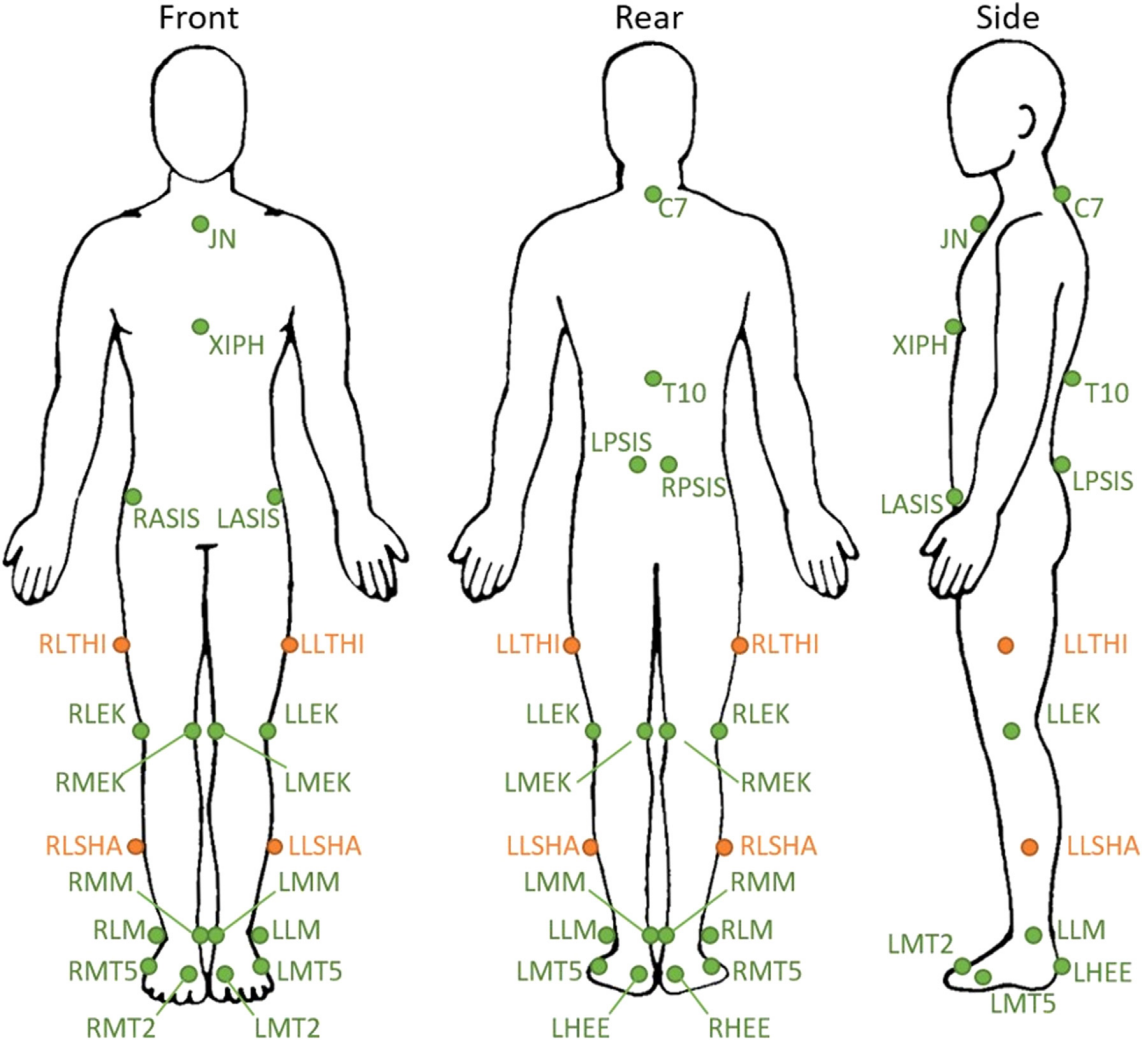
Front, side and rear view of the marker set used in human body model lower limb with trunk markers. JN, jugular notch of the sternum; C7, 7th cervical vertebra; XIPH, xiphoid process of the sternum; T10, 10th thoracic vertebra; RASIS/LASIS, right/left anterior superior iliac spine; RPSIS/LPSIS, right/left posterior superior iliac spine; RLTHI/LLTHI, right/left lateral thigh; RLEK/LLEK, right/left lateral epicondyle of the knee; RMEK/LMEK, right/left medial epicondyle of the knee; RLSHA/LLSHA, right/left lateral shank; RMM/LMM, right/left medial malleolus of the ankle; RLM/LL, right/left lateral malleolus of the ankle; RMT5/LMT5, caput of the 5th meta tarsal bone; RMT2/LMT2, right/left caput of the 2nd meta tarsal bone; RHEE/LHEE, right/left heel (same height as LMT).

Three-dimensional gait analysis was conducted at the Computer Assisted Rehabilitation ENvironment (CAREN, Motek Medical BV) system. CAREN includes a dual-belt instrumented treadmill (force plates: 1000Hz), a 12-camera motion capture system (100Hz; Vicon Motion Systems) and a virtual industrial environment providing optic flow. All participants wore standard gymnastic shoes and a safety harness connected to an overhead frame to prevent falling. Both patients and controls followed the same protocol, which has been described at protocols.io [25]. Twenty-six reflective markers attached directly onto the skin at specific bony landmarks according to the Human Body lower limb model with trunk markers (HBM-II) were tracked by the motion capture system (Fig. 1). All subjects walked at their individual comfortable speed. To determine the comfortable walking speed, subjects started to walk at 0.5 m/s and walking speed was increased every second with 0.01 m/s until the subjects stated that their comfortable walking speed was reached. This was repeated 3 times and the average was taken as the comfortable walking speed. After a 6-minute familiarization period, a total of 250 steps (125 cycles) were recorded.

#### Biplanar radiographic exam

Static alignment was analyzed using standardized anteriorposterior and lateral full-spine radiographs using validated software (Surgimap; Nemaris). The following radiographic parameters were measured: maximal coronal Cobb angle (°), pelvic tilt (PT,°), pelvic incidence (PI,°), sacral slope (SS,°), L1–S1 lumbar lordosis (L1–S1 LL,°), L4–S1 lumbar lordosis (L4–S1 LL,°), pelvic incidence—lumbar lordosis (PI–LL) mismatch (°), thoracic kyphosis (TK,°), global tilt (GT,°) and sagittal vertical axis (SVA; mm). The Global Alignment and Proportion score was also calculated [26].

### Outcomes

Data recording and processing have been described in a paper at protocols.io [25]. The quality of the data was checked, and good kinematic and kinetic steps were identified using custom-made algorithms programmed in Matlab (R2016a, mathworks). The following spatiotemporal parameters were determined based on all valid steps: walking speed (m/s), cadence (steps/min), stride length (m), stride time (s), stance time (s), swing time (s), double support time (s), and step width (m). The coefficient of variation (CoV, [standard deviation / mean]*100) of the stride time (%) and stride length (%) was calculated as a measure of variability. Data of joint kinematics were time normalized (gait cycle 0%–100%). For every time point of the gait cycle, the average over all valid steps was calculated per subject. The following joint kinematics were calculated: sagittal, frontal and transversal plane trunk and pelvis joint angles (˚) and sagittal hip, knee and ankle angle (˚). In addition, range of motion (ROM) in joint kinematics was calculated as maximum–minimum value over the whole gait cycle. Subsequently, group averages for the ID-ASD patient group, DN-ASD patient group and both control groups were calculated.

### Statistics

Normality of demographic data, spatiotemporal and CoV parameters and ROM in joint kinematics data was tested with Shapiro—Wilk test and reported as median and Q1 to Q3. Depending on the normality of data, the independent t-test or Mann—Whitney U test was used to compare spatiotemporal—and CoV parameters and ROM in joint kinematics of both patient groups with their control group. Statistical parametric mapping (SPM), using 2-tailed 2-sample t-test, was used to compare the joint kinematics of the patient versus control groups, and the patients with ID-ASD versus patients with DN-ASD [27]. Joint kinematics waveforms were presented as group average with standard deviations. The analyses were done using the Statistical Package for the Social Sciences version 25 (SPSS) and SPM analyses were implemented using the open-source spm1d code (v.M0.4.7 (2019.11.27), www.spm1d.org) in MATLAB (Mathworks, R2016a) [28]. Significance level was set for all analyses at p < .05.

## Results

### Demographics

Demographics of the symptomatic idiopathic scoliosis (ID-ASD) group (n = 24), “de novo” scoliosis (DN-ASD) group (n = 26) and their own matched control group (N = 50) are shown in Table 1. Age, gender, weight, leg length, height and Body Mass Index (BMI, weight*height^2^) were comparable between the patient groups and their control groups. As anticipated, patients with ID-ASD were significantly younger compared with patients with DN-ASD, and had significantly lower BMI.

**Table 1:** Demographics.

	ID-ASD			Controls			ID-ASD vs C	DN-ASD			Controls			DN-ASD vs C	ID-ASD vs. DN-ASD
	Median	Q1	Q3	Median	Q1	Q3	p-value	Median	Q1	Q3	Median	Q1	Q3	p-value	p-value
Gender (F/M)	15/9	-	-	15/9	-	-	-	21/5	-	-	21/5	-	-	-	-
Age (y)	20.0	19.0	26.5	22.0	20.3	30.0	.119	60.5	55.0	65.5	59.5	51.5	66.3	.558	< .001*
Weight (kg)	68.5	58.3	75.4	70.3	62.3	76.8	.571	75.5	64.9	87.3	73.4	66.1	82.1	.616	.061
Height (m)	1.71	1.64	1.78	1.73	1.68	1.78	.371	1.65	1.58	1.73	1.66	1.61	1.75	.782	.126
BMI (kg/m^2^)	23.1	20.7	26.7	22.3	20.9	25.6	.934	28.1	25.1	30.1	27.3	23.9	28.7	.477	.006*
Leg length (m)	0.90	0.85	0.93	0.89	0.86	0.94	.898	0.89	0.83	0.93	0.87	0.84	0.92	.729	.176
Total hip replacements	0	-	-	0	-	-	-	1 (bilateral)	-	-	0	-	-	-	-
Total knee replacements	0	-	-	0	-	-	-	1	-	-	0	-	-	-	-

Gender is reported as absolute numbers. Age, weight, height BMI and leg length is reported as medians and first and third quartile.

⁎ Significance level: p < .05. ID-ASD, symptomatic idiopathic scoliosis-adult spinal deformity; DN-ASD, “de novo” scoliosis-adult spinal deformity; C, controls.

### Radiographic parameters

**Fig. 2:**
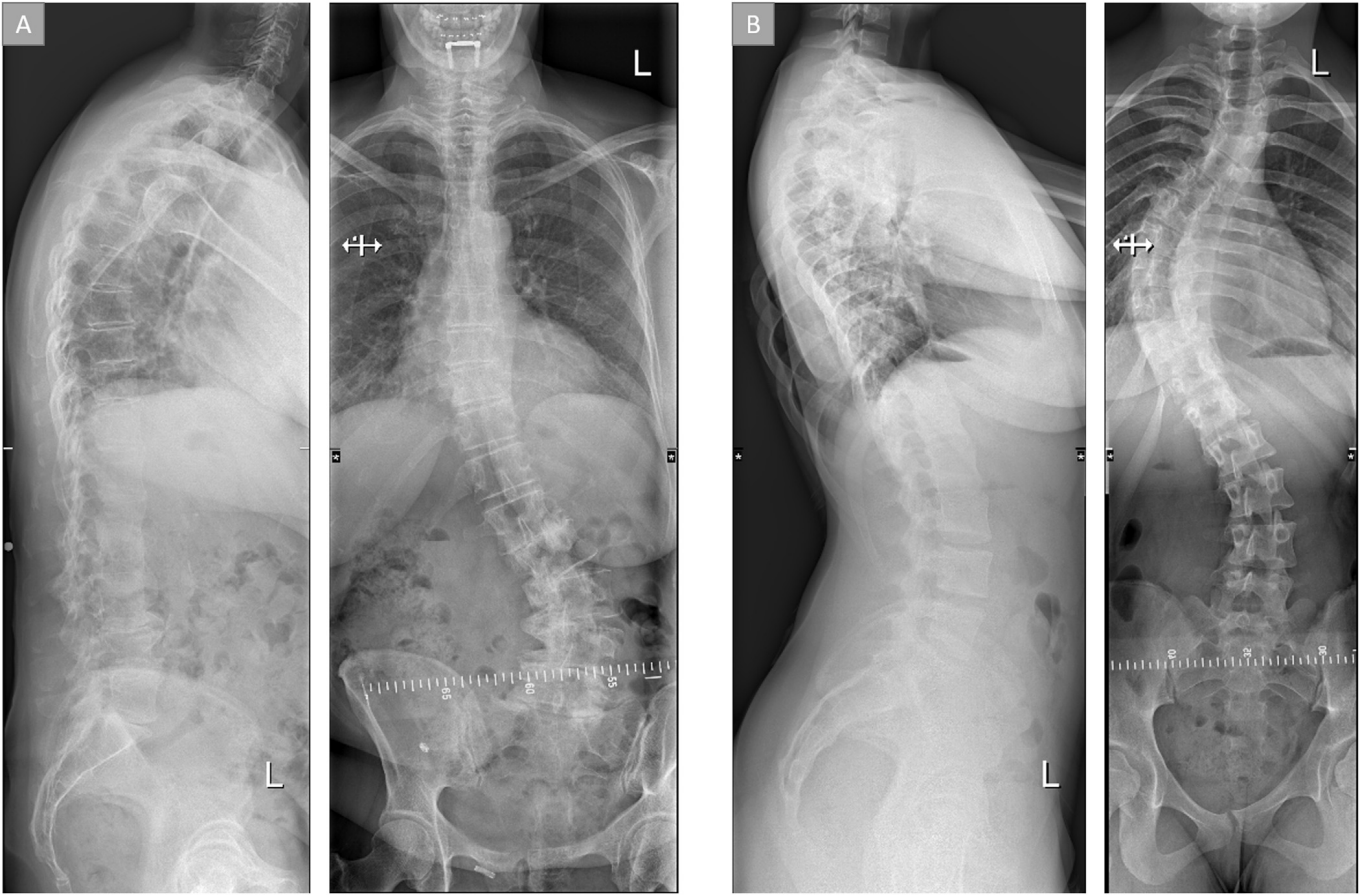
Typical radiographic images of ASD patients. (A) "de novo" ASD patient, (B) symptomatic idiopathic scoliosis ASD patient.

Radiographic parameters of the ID-ASD group and DN-ASD group are shown in Table 2 and 2 exemplary radiographs are shown in Fig. 2. In the coronal plane, a significant difference in Cobb angle between the ID-ASD and the DN-ASD group was found with the ID-ASD patients displaying a larger Cobb angle compared with the DN-ASD group (Δ17°). With regards to radiographic parameters in the sagittal plane increased anterior pelvic tilt (Δ11°), and a larger pelvic incidence (Δ13.5°), global tilt (Δ19.0°) and sagittal vertical axis (Δ68mm) was found for the DN-ASD group compared with the ID-ASD group. Furthermore, the DN-ASD group had a significantly larger Global Alignment and Proportion score compared with the ID-ASD group (7.0 [3–10] vs. 2.0 [0–5]).

**Table 2:** Radiographic parameters.

	ID-ASD			DN-ASD			ID-ASD vs. DN-ASD
	Median	Q1	Q3	Median	Q1	Q3	p-value
Maximal coronal Cobb angle (°)	46.00	36.50	54.00	29.00	15.25	42.75	.003*
Pelvic tilt (°)	13.00	7.00	18.00	24.00	17.75	33.00	< .001*
Pelvic incidence (°)	42.00	38.00	56.00	55.5	42.5	63.75	.006*
Sacral slope (°)	33.00	28.00	42.00	31.5	22.00	39.25	.792
Lumbar lordosis L1S1 (°)	52.00	41.00	60.00	39.5	28.25	57.00	.099
Lumbar lordosis L4S1 (°)	33.00	29.00	40.00	32.50	21.00	38.25	.313
Pelvic incidence – Lumbar lordosis mismatch (°)	11.00	6.00	20.00	17.00	3.75	27.50	.176
Thoracic kyphosis (°)	39.00	20.00	49.00	42.00	29.50	54.25	.307
Global tilt (°)	9.00	5.00	22.00	28.00	24.00	42.00	< .001*
Sagittal vertical axis (mm)	20.00	0.00	29.00	88.00	40.00	131.00	< .001*
GAP score	2.00	0.00	5.00	7.00	3.00	10.00	.001*

Data is reported as medians and interquartile ranges.

⁎ Significance level: p < .05. Not all parameters were available for all patients: ID-ASD maximal coronal Cobb angle n = 21; ID-ASD sagittal parameters n = 23; DN-ASD maximal coronal Cobb angle n = 18; DN-ASD sagittal parameters n = 26. ID-ASD, symptomatic idiopathic scoliosis-adult spinal deformity; DN-ASD, “de novo” scoliosis-adult spinal deformity.

### Joint kinematics

**Fig. 3:**
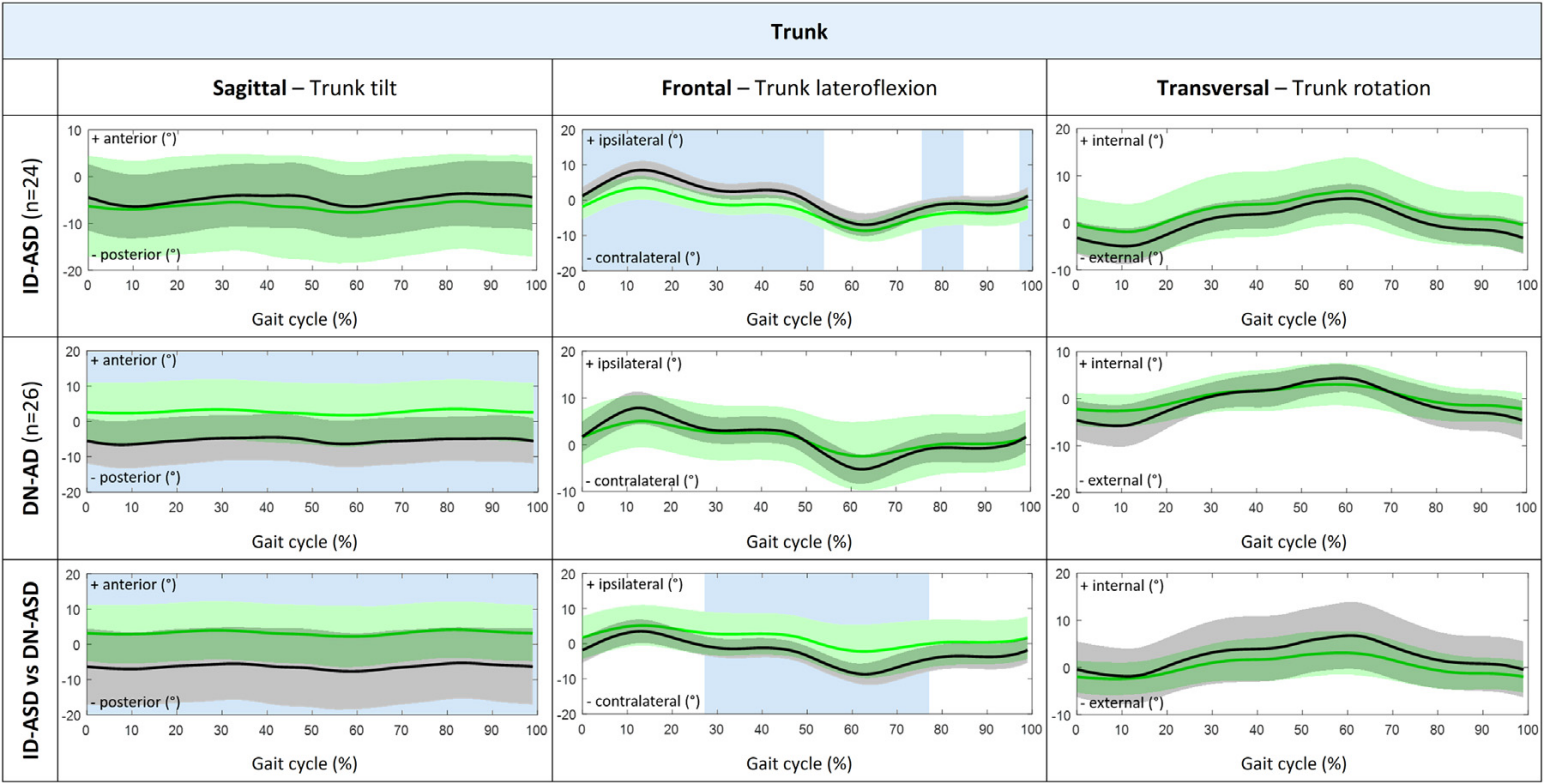
3D Trunk kinematic waveforms presented as group average with standard deviations for trunk tilt, trunk lateroflexion and trunk rotation during complete gait cycle (0%–100%). Comparison between patients with ID-ASD (green) compared with controls (black) in the first row, patients with DN-ASD (green) compared with controls (black) in the second row and patients with ID-ASD (black) compared with patients with DN-ASD (green) in the third row. The blue shaded areas indicate the part of the gait cycle (%) were the kinematic waveforms significantly differ between groups tested with SPM 2-tailed 2-sample t-test. 3D, three-dimensional; ID-ASD, symptomatic idiopathic scoliosis-adult spinal deformity; DN-ASD, "de novo"-adult spinal deformity; SPM, statistical parametric mapping.

**Fig. 4:**
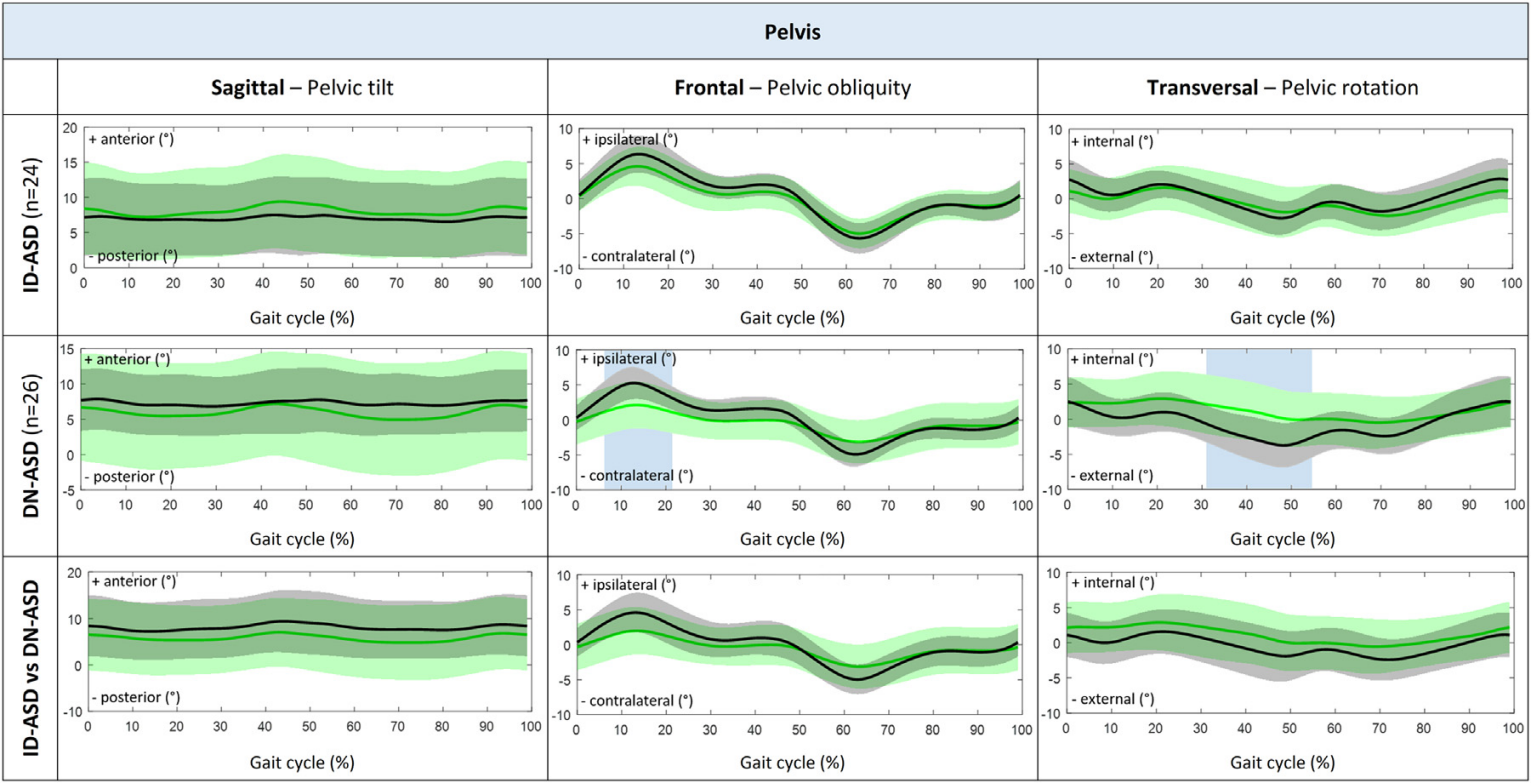
3D Pelvic kinematic waveforms presented as group average with standard deviations for pelvic tilt, pelvic obliquity and pelvic rotation during complete gait cycle (0%–100%). Comparison between patients with ID-ASD (green) compared with controls (black) in the first row, patients with DN-ASD (green) compared with controls (black) in the second row and patients with ID-ASD (black) compared with patients with DN-ASD (green) in the third row. The blue shaded areas indicate the part of the gait cycle (%) were the kinematic waveforms significantly differ between groups tested with SPM 2-tailed 2-sample t-test. 3D, three-dimensional; ID-ASD, symptomatic idiopathic scoliosis-adult spinal deformity; DN-ASD, "de novo"-adult spinal deformity; SPM, statistical parametric mapping.

**Fig. 5:**
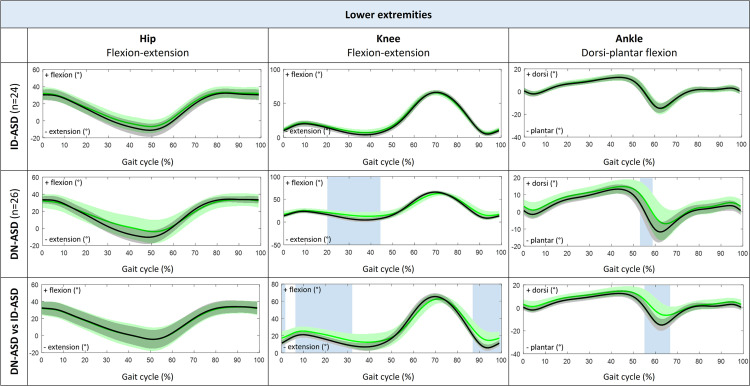
Sagittal hip, knee and ankle kinematic waveforms presented as group average with standard deviations for pelvic tilt, pelvic obliquity and pelvic rotation during complete gait cycle (0%–100%). Comparison between patients with ID-ASD (green) compared with controls (black) in the first row, patients with DN-ASD (green) compared with controls (black) in the second row and patients with ID-ASD (black) compared with patients with DN-ASD (green) in the third row. The blue shaded areas indicate the part of the gait cycle (%) were the kinematic waveforms significantly differ between groups tested with SPM 2-tailed 2-sample t-test. 3D, three-dimensional; ID-ASD, symptomatic idiopathic scoliosis-adult spinal deformity; DN-ASD, "de novo"-adult spinal deformity; SPM, statistical parametric mapping.

Patients with ID-ASD walked with significant less trunk lateroflexion during stance phase and midswing, while trunk tilt, trunk rotation and 3D pelvic motion was comparable to their control group (Figs. 3 and 4). In addition, the ID-ASD group showed similar hip, knee and ankle joint kinematics compared with controls (Fig. 5).

Patients with DN-ASD showed significantly increased anterior trunk tilt during the whole gait cycle, decreased pelvic obliquity during loading response and decreased external pelvic rotation from midstance to preswing as compared with their controls. Conversely, no differences were found in trunk lateroflexion, trunk rotation or pelvic tilt (Figs. 3 and 4). Besides, the DN-ASD group had significantly increased knee flexion from midstance to preswing and decreased ankle plantar flexion during preswing compared with their controls (Fig. 5).

When comparing ID-ASD patients with DN-ASD patients, patients with DN-ASD walked with significantly increased anterior trunk tilt, decreased trunk lateroflexion from midstance until midswing, while trunk rotation and 3D pelvic motion were comparable to the patients with ID-ASD (Figs. 3 and 4). Further, the DN-ASD group displayed decreased knee extension from terminal swing until midstance with significantly decreased plantar flexion during preswing (55%–66% of gait cycle).

### Range of motion

Patients with ID-ASD showed significantly decreased ROM in trunk lateroflexion (Δ3.04°), significantly increased ROM in pelvic tilt (Δ0.71°), and significantly decreased pelvic obliquity (Δ1.87°) and hip flexion/extension (Δ3.45°) compared with their controls (Table 3). Patients with DN-ASD displayed significantly decreased ROM in trunk rotation (Δ3.58°), significantly increased ROM in pelvic tilt (Δ1.41°), significantly decreased pelvic obliquity (Δ5.04°), pelvic rotation (Δ1.58°), hip flexion/extension (Δ6.33°) and knee flexion/extension (Δ7.45°) (Table 3). Compared with patients with ID-ASD, patients with DN-ASD showed significantly decreased ROM in trunk lateroflexion (Δ4.07°), trunk rotation (Δ3.40°), pelvic obliquity (Δ4.57°) and knee flexion/extension (Δ6.40°) (Table 3).

**Table 3:** Range of motion during walking.

	ID-ASD			Controls			ID v C	DN-ASD			Controls			DN v C	ID v DN
	Median	Q1	Q3	Median	Q1	Q3	p-value	Median	Q1	Q3	Median	Q1	Q3	p-value	p-value
ROM trunk tilt (°)	3.70	2.86	4.68	3.95	3.46	5.22	.213	3.14	2.41	3.77	3.21	2.50	4.16	.464	.081
ROM trunk lateroflexion (°)	12.42	9.63	15.06	15.46	12.27	18.64	.024*	8.35	5.36	10.29	10.50	7.08	13.74	.052	< .001*
ROM trunk rotation (°)	9.56	6.73	11.27	10.17	7.99	12.90	.098	6.16	4.11	8.26	9.74	7.51	14.08	< .001*	.013*
ROM pelvic tilt (°)	2.94	2.11	4.06	2.13	1.76	2.38	< .001*	3.91	2.87	4.49	2.50	2.06	2.82	< .001*	.107
ROM pelvic obliquity (°)	10.22	7.37	12.53	12.09	11.11	13.87	.011*	5.65	4.27	8.39	10.69	8.14	12.15	< .001*	< .001*
ROM pelvic rotation (°)	6.27	4.59	7.83	6.75	5.29	8.88	.213	4.74	3.84	7.33	6.32	3.79	9.90	.234	.251
ROM hip flexion/extension (°)	41.60	36.65	44.74	43.43	41.48	48.37	.008*	39.81	32.95	43.81	46.17	41.00	49.74	.002*	.254
ROM knee flexion/extension (°)	62.56	57.09	65.91	63.29	61.35	66.49	.089	56.16	48.88	59.86	63.61	57.81	65.86	< .001*	< .001*
ROM ankle dorsi/plantar flexion (°)	29.05	26.62	31.87	28.40	24.69	31.09	.771	25.76	19.74	30.26	27.46	22.56	29.10	.689	.122

Medians and interquartile ranges are reported.

⁎ Significance level: p < .05. ROM, range of motion; ID-ASD, symptomatic idiopathic scoliosis-adult spinal deformity; DN-ASD, “de novo”-adult spinal deformity; C, controls.

### Spatiotemporal parameters

Spatiotemporal parameters (Table 4) were comparable for patients with ID-ASD and their control group. Patients with DN-ASD walked significantly slower (Δ0.31m/s) with decreased cadence (Δ10steps/min) and stride length (Δ0.21m) and increased stride time (Δ0.09s), stance time (0.09s), double support time (0.03s) and step width (0.04m) compared with controls. Furthermore, patients with DN-ASD walked significantly slower (Δ0.18m/s) with decreased stride length (Δ0.17m), stance time (Δ0.04s) and double support time (Δ0.02s) compared with patients with ID-ASD.

**Table 4:** Spatiotemporal parameters during walking.

	ID-ASD			Controls			ID v C	DN-ASD			Controls			DN v C	ID v DN
	Median	Q1	Q3	Median	Q1	Q3	p-value	Median	Q1	Q3	Median	Q1	Q3	p-value	p-value
Speed (m/s)	1.17	1.04	1.28	1.21	1.09	1.34	.210	0.99	0.73	1.14	1.30	1.13	1.39	< .001*	.004*
Cadence (steps/min)	112.76	101.18	118.54	110.9	104.7	115.2	.683	108.40	101.83	113.25	118.24	111.32	122.79	.001*	.400
Stride length (m)	1.25	1.16	1.37	1.31	1.17	1.49	.186	1.08	0.84	1.28	1.29	1.21	1.37	< .001*	< .001*
Stride time (s)	1.07	1.01	1.19	1.08	1.04	1.15	.967	1.11	1.07	1.18	1.02	0.98	1.08	.001*	.312
Stance time (s)	0.66	0.60	0.73	0.67	0.62	0.68	.773	0.70	0.66	0.76	0.61	0.58	0.66	< .001*	.047*
Swing time (s)	0.42	0.41	0.46	0.43	0.42	0.45	.379	0.42	0.40	0.44	0.41	0.38	0.43	.399	.572
Double support time (s)	0.12	0.10	0.13	0.11	0.10	0.12	.241	0.14	0.12	0.17	0.11	0.09	0.13	< .001*	.002*
Step width (m)	0.18	0.14	0.20	0.18	0.13	0.21	.606	0.20	0.18	0.24	0.16	0.14	0.20	.024*	.193

Medians and interquartile ranges are reported.

⁎ Significance level: p < .05. ID-ASD, symptomatic idiopathic scoliosis-adult spinal deformity; DN-ASD, “de novo”-adult spinal deformity; C, controls.

### Gait variability (CoV)

With regards to gait variability, the ID-ASD group walked with comparable stride time variability (1.81 [1.29–2.00]% vs. 1.72[1.01–1.89]%) and stride length variability (2.13 [1.56–2.63]% vs. 1.92 [1.48–2.38]%) to their controls. The patients with DN-ASD showed increased variability in stride time (2.00 [1.78–3.07]% vs. 1.52 [1.01–1.85]%) and stride length (2.92[2.19-4.39]% vs. 1.72[1.49–2.61]%) compared with controls and compared with patients with ID-ASD (Δ0.19%; Δ0.79% resp.).

## Discussion

Identifying and characterizing gait alterations caused by ASD is crucial for evaluating the functional impact of spinal deformity on a patient's daily mobility, designing interventions customized to their specific needs, and monitoring treatment progress effectively. The current study aimed to compare gait characteristics between ASD patients with symptomatic idiopathic scoliosis (ID-ASD) and ASD patients with “de novo” scoliosis (DN-ASD) scheduled for spinal fusion, along with matched asymptomatic healthy controls. The results reveal that patients with DN-ASD exhibited greater alterations in spatiotemporal and kinematic gait parameters compared with controls, as well as in comparison to patients with ID-ASD. Moreover, patients with DN-ASD show increased variability in stride time and stride length.

During the whole gait cycle, DN-ASD patients walk with significantly increased anterior trunk tilt, which is in line with previous studies on patients with ASD [3,6,18,29]. The observed increase in anterior trunk tilt indicates sagittal imbalance and might be attributed to spinal malalignment in the sagittal place, which corresponds with the radiographic finding showing a significantly SVA and global tilt for DN-ASD patients compared with ID-ASD patients. It suggests a relation between SVA and global tilt with trunk tilt waveforms, which is confirmed with additional analysis (Appendix). This sagittal imbalance may also arise from increased stiffness of the spine and can be a consequence from limited pelvic obliquity and pelvic rotation due to weakness of hip abductors, general deconditioning and lumbar deformity [[28], [29], [30], [31]].

In contrast, the patients with ID-ASD do not show deviating trunk tilt, but do show significantly reduced trunk lateroflexion during stance. This corresponds with the static radiographs, where the ID-ASD group had significant smaller SVA and global tilt compared with the DN-ASD groups. This is in line with Karam et al. [23], who states that patients with ID-ASD often display postural malalignment in the frontal plane, but normal alignment in the sagittal plane as shown by static radiographs. Semaan et al. [29] also found that when scoliosis is limited to the frontal plane, it does not impact sagittal trunk tilt during walking. Interestingly, additional analyses (Appendix) showed no significant positive correlation between maximal coronal Cobb angle and trunk lateroflexion waveforms. But, is has previously been shown in AIS patients that a scoliosis can lead to asymmetric trunk movement in the frontal plane, where patients lean towards either side, depending on the nature of the spinal deformity [32]. This asymmetry in trunk lateroflexion means that part of the ID-ASD group may display increased trunk lateroflexion in one direction, while another part of the ID-ASD group may have increased trunk lateroflexion in the opposite direction. This difference in these trunk movements may partially cancel each other out, leading to a reduced net trunk lateroflexion on average.

Despite a significant increased static radiographic pelvic tilt in the DN-ASD patients compared with the ID-ASD patients, no significant differences are found in pelvic tilt during walking neither between ID-ASD patients and DN-patients, nor when compared with their control groups which is in line with prior investigations [3,4,12,19,20,29,33,34]. Moreover, the significant positive correlation between trunk tilt during walking and the static radiographic pelvic tilt highlights that DN-ASD patients experience more sagittal spinal malalignment compared with ID-ASD while standing (Appendix). And, as the dynamic pelvic tilt is comparable to controls, that this compensation mechanism of pelvic retroversion during standing in DN-ASD patients is lost during walking [[3], [4], [5], [6], [7]]. In addition, ASD patients demonstrate a larger standard deviation in pelvic tilt kinematics when compared with controls, signifying interindividual variations in pelvic tilt during walking [12,29]. Both ID-ASD and DN-ASD patients exhibit a significantly increased ROM in pelvic tilt than controls, suggesting increased pelvic motion in the sagittal plane on individual level. This may be attributed to the need to minimize the center of mass excursion [6,35], or due to the loss of compensatory mechanisms aimed at maintaining sagittal balance during walking. A study of Bae et al. [36] investigated the impact of fatigue on compensatory mechanisms during walking in patients with ASD. Their findings reveal that, following 10 minutes of walking, 84.6% of ASD patients with compensated sagittal deformity before walking lose the capacity to compensate through pelvic retroversion or thoracic hyperextension. Consequently, sagittal misbalance occurred. In the current study, participants were required to walk for a prolonged duration, lasting up to 12 minutes, encompassing both the familiarization period and the measuring period. Although not investigated in the current study, it is plausible that, similar to the patients in the study of Bae et al. [36], ASD patients are able to compensate at the beginning of the measurements but lose this ability at the end of the measurement. This may partly explain the larger standard deviation in pelvic tilt kinematics and pelvic tilt ROM in the ASD patient groups, especially in the DN-ASD group who are expected to experience more pronounced deviations in the sagittal plane and more progressive muscle fatigue with activity, general deconditioning and secondary pain [5,7,24,37].

Regarding lower extremity kinematics, patients with DN-ASD exhibit decreased knee extension during stance and decreased ankle plantar flexion around toe off compared with controls, while ID-ASD patients show decreased hip extension, accounting for the decreased ROM in both hip and knee. These findings are consistent with a crouched gait pattern, which has been previously reported in patients with ASD as a compensatory mechanism to keep the center of gravity above the feet and remain a horizontal gaze during walking [3,5,6,7,29].

Patients with ID-ASD show similar spatiotemporal gait parameters compared with their controls, suggesting that the spinal deformation for these patients does not yet impose a significant problem concerning ambulation. A study by Semaan et al. [29] evaluated differences in ASD patients with different types of spinal deformity and also found that spatiotemporal parameters of ASD patients with a spinal deformity in the frontal plane were similar to controls. In contrast, DN-ASD patients walk with slower walking speed compared with controls, and with smaller and wider steps with increased double support time. This might be a compensation mechanism to improve stability during walking [6,12,29], as the sagittal deformation challenges the sagittal balance. Furthermore, we found that DN-ASD patients show increased gait variability compared with controls. The increased stride time and stride length variability in combination with slower walking speed in DN-ASD patients indicates a less consistent and less balanced gait [6,38,39]. This is in line with results found by Simon et al. [13] who showed significantly less step length consistency in patients with ASD compared with controls. Also, increased variability in stride time and stride length have previously been identified as fall predictors in patients with gait and balance problems, suggesting that the DN-ASD group experiences sagittal imbalance and may be at a higher risk for falling than their control group [13,29,[38], [39], [40], [41]].

The study has some limitations that warrant consideration. First, patients walked at a slower comfortable walking speed compared with controls, which may have influenced spatiotemporal parameters and joint kinematics [42]. However, this slower speed is a study finding in itself, and using a fixed walking speed would have potentially forced subjects to walk at an uncomfortable pace, potentially leading to an unnatural gait pattern. Second, this study examined trunk motion as 1 rigid segment, which limits the ability to gather information on the curvature of the spine or kinematic changes within the spine [3,[43], [44], [45],^46^]. Third, no radiographic images were available for the controls, which made it impossible to compare radiographic parameters of ASD patients with their matched controls. Last, although it is known that musculoskeletal conditions such as osteoarthritis or neurological disorders such as Parkinson's disease may influence gait patterns, we did not take this into consideration in the current study [47].

This study boasts several notable strengths. Unlike common presentation of kinematic parameters as peak values and/or range of motion [12,17,20], the utilization of SPM analyses enables a comprehensive comparison of joint kinematics throughout the entire gait cycle, offering deeper insights into the gait characteristics of patients with ASD. Moreover, 3-dimensional gait analysis conducted on a treadmill system allows for the measurement of numerous successive steps, resulting in a large dataset and increased data reliability [48]. Conversely, overground trials are constrained by limited walking distance, which restricts the number of recorded successive steps. The acquisition of many successive steps not only facilitates the assessment of gait variability but would also permit the detection of compensatory mechanism failure by fatigue, a task that may not be feasible in short-distance tracks [24]. Additionally, while many studies tend to report only kinematics in the sagittal plane, this study encompasses kinematic data from 3-dimensional planes, providing a more comprehensive perspective on gait characteristics. Last, the meticulous matching of each patient to an age, sex, leg length, and BMI-matched control individual ensures that any differences found in gait characteristics are not influenced by these demographic variables.

## Conclusion

This study demonstrates that ASD patients with “de novo” scoliosis (DN-ASD) exhibit distinct alterations in spatiotemporal and kinematic gait characteristics compared with ASD patients with symptomatic idiopathic scoliosis (ID-ASD) and compared with asymptomatic controls. Specifically, patients with ID-ASD display limited trunk lateroflexion and hip extension during stance, whereas DN-ASD patients exhibit a slower walking speed and corresponding changes in spatiotemporal parameters, accompanied by increased anterior trunk tilt, limited pelvic obliquity and rotation, and limited knee extension during stance. These alterations seem to be predominantly influenced by sagittal spinal malalignment. These findings emphasize the significance of taking into consideration the nature of spinal deformity in ASD patients as it may have a different effect on daily functioning and therefore overall quality of life.

## Declarations of Competing Interest

The authors declare that they have no known competing financial interests or personal relationships that could have appeared to influence the work reported in this paper.

## Appendix Supplementary materials

Image, application 1
