# Supplementary material for: Gait alterations in patients with adult spinal deformity
Source: N Am Spine Soc J. 2023 Dec 30;17:100306. doi: 10.1016/j.xnsj.2023.100306 (PMC10825775; doi:10.1016/j.xnsj.2023.100306)
Supplement: Supplementary file 1 [file mmc1.docx]

**Appendix – SPM regression analysis**

| **Maximal coronal Cobb angle vs:** | |
| --- | --- |
| Trunk tilt | Trunk lateroflexion |
|  |  |
| **Pelvic tilt vs:** | |
| Trunk tilt vs pelvic tilt | Trunk lateroflexion vs pelvic tilt |
|  |  |
| **Pelvic incidence vs:** | |
| Trunk tilt | Trunk lateroflexion |
|  |  |

| **Sagittal vertical axis vs:** | |
| --- | --- |
| Trunk tilt | Trunk lateroflexion |
|  |  |
| **GAP score vs:** | |
| Trunk tilt | Trunk lateroflexion |
|  |  |

***Figure appendix.*** *SPM regression analysis with all patients data (n=50) were used to correlate trunk tilt and trunk lateroflexion waveforms (0-100% of the gait cycle) to coronal maximal Cobb angle, pelvic tilt (PT), pelvic incidence (PI), sagittal vertical angle (SVA) and GAP score. The grey shaded areas indicate the part of the gait cycle (%) were the kinematic waveforms and radiographic parameter significantly correlate. SPM = statistical parametric mapping.*
